# Vick's Floral Guide for 1886

**Published:** 1886-02

**Authors:** 


VICK'S FLORAL GUIDE FOR 1886.—The Illustrated Holliday number is by far the handsomest of these annuals yet issued by the reliable and widely known house of James Vick, Rochester, New York. This number contains 160 pages profusely illustrated with the addition of two finely executed full page chromo lithographs. Besides the descriptive catalogues, which are very complete, over thirty pages are devoted to instruction in horticulture and familiar talks, which render the volume both interesting and useful.

